# *PTPN22 *polymorphisms may indicate a role for this gene in atopic dermatitis in West Highland white terriers

**DOI:** 10.1186/1756-0500-4-571

**Published:** 2011-12-30

**Authors:** Joana Barros Roque, Caroline A O'Leary, Myat Kyaw-Tanner, David L Duffy, Puya Gharahkhani, Linda Vogelnest, Kenneth Mason, Michael Shipstone

**Affiliations:** 1 School of Veterinary Science, The University of Queensland, Gatton, Queensland, 4343, Australia; 2 Centre for Companion Animal Health, School of Veterinary Science, The University of Queensland, St Lucia, Queensland, 4069, Australia; 3 Genetic Epidemiology Laboratory, Queensland Institute of Medical Research, Herston, Queensland, 4029, Australia; 4 The University of Sydney, University Veterinary Teaching Hospital, Camden, New South Wales, 2570, Australia; 5 Dermcare, Springwood, Queensland, 4127, Australia; 6 Dermatology for Animals, Stafford Heights, Queensland, 4053, Australia

## Abstract

**Background:**

Canine atopic dermatitis is an allergic inflammatory skin disease common in West Highland white terriers. A genome-wide association study for atopic dermatitis in a population of West Highland white terriers identified a 1.3 Mb area of association on CFA17 containing canine protein tyrosine phosphatase non-receptor type 22 (lymphoid) *PTPN22*. This gene is a potential candidate gene for canine atopic dermatitis as it encodes a lymphoid-specific signalling mediator that regulates T-cell and possibly B-cell activity.

**Findings:**

Sequencing of *PTPN22 *in three atopic and three non-atopic West Highland white terriers identified 18 polymorphisms, including five genetic variants with a bioinformatically predicted functional effect. An intronic polymorphic repeat sequence variant was excluded as the cause of the genome-wide association study peak signal, by large-scale genotyping in 72 West Highland white terriers (gene-dropping simulation method, *P *= 0.01).

**Conclusions:**

This study identified 18 genetic variants in *PTPN22 *that might be associated with atopic dermatitis in West Highland white terriers. This preliminary data may direct further study on the role of *PTPN22 *in this disease. Large scale genotyping and complementary genomic and proteomic assays would be required to assess this possibility.

## Findings

Canine atopic dermatitis (AD) is an allergic inflammatory skin disease that is common in West Highland white terriers (WHWTs) [1]. Following a genome-wide association (GWAS) in a group of related WHWTs, we found a 1.3 Mb area on CFA 17 which was significantly associated with the disease [2]. Based on its biological functions, expression patterns and proximity to this area of association, *PTPN22 *was selected as a candidate gene for AD in this population. This gene encodes a lymphoid tyrosine phosphatase (PTPN22), a signalling mediator that regulates generic and specialised immune functions in mammals [3]. Activation of T and B lymphocytes is a key event in the pathogenesis of atopic disease [4], and the disruption of these pathways could cause hyper-reactive pathogenic T-cell responses, as well as affect B-cell selection, maturation and function [5,6]. In humans and dogs, genetic variants in the gene *PTPN22 *have been associated with auto-immune diseases [7-9]. In humans, these include psoriasis, a chronic immune-mediated inflammatory skin disease that shares susceptibility loci with human AD [10,11]. To date, no association has been found between *PTPN22 *variants and atopic disease in humans [12].

The University of Queensland Animal and Human Ethics Committees, and the University of Sydney Animal Ethics Committee approved this study. Written consent was obtained from all participating dog owners.

Criteria used to classify dogs in the present study are described elsewhere [1]. Fourteen set of primers were designed with primer3 [13], to sequence a total of 12.6 Kb of *PTPN22 *in 14 PCR products (Table 1). Amplification reactions used the HotStar HiFidelity PCR Kit (QIAGEN Pty Ltd, Doncaster, Vic, Australia) and 0.5 μM (PCR products 5 and 12), 1.5 μM (6 and 14) or 1 μM (remaining PCR products) of primers; at 55°C (PCR product 8), 57°C (3 and 14), 58°C (7, 10 and 13), 64°C (5) or 60°C (remaining products) annealing temperatures. PCR products were purified with MinElute PCR Purification Kit (QIAGEN Pty Ltd, Doncaster, Vic, Australia), and bi-directionally sequenced at the Australian Equine Genetics Research Centre using 0.5 μM (PCR product 3, 4, 5, 12, 14) or 1 μM (remaining PCR products) of forward and reverse amplification primers and 0.5 μM of internal sequencing primers (Table 1), and BigDye Terminator v3.1 Cycle Sequencing Kit (Applied Biosystems, Foster City, CA, USA). Primers were supplied by GeneWorks (Hindmarsh, SA, Australia). Sequencing protocol was as recommended by the manufacturer, except annealing temperatures for PCR products 3, 9 and 11 were 50°C and 60°C for PCR products 4 and 5.

**Table 1:** Primer sequences used to amplify and sequence 12.6 Kb of canine *PTPN22 *in three atopic and three non-atopic WHWTs

PCR product	Forward amplification primer	Reverse amplification primer	Internal forward sequencing primer	Internal reverse sequencing primer	Predicted gene region	Product size
**1**	CCTCATCAGGTGCTCTTCGT	GGTTTTGCCTCTCTCCCTTC	TGAAGTGGAAGAGTCTCAGAGC	AGAAAAGGCAGAAGGCCAGT	5'UTR, exon 1	1041

**2**	GGCTCTGTCCTGAATTGGAG	TCTGCCCTTACCAGGACACT	-	-	Exons 2,3	858

**3**	CCAAATAAGAGGTCGGGGTA	CTACTGGGAAAATGGGCAAA	AGAAAAGGGAAGGAAGGACA	TCTGTCCTTCCTTCCCTTTTC	Exons 4,5	863

**4**	ACCACAGTTGACCTTGGATAA	AGATGAAGGCACATCATGGTC	-	ACATCAAAGGTCCCCTACTCC	Exons 6,7	1182

**5**	CCACTTGAACTGGTGAAGCA	ACCAGTCCTTCCACAACCAG	-	GGATGGAACCCCATATTGAA	Exons 8	1172

**6**	TGCTCTGGGAAGTAGGGATG	CAAGGCAAGGGACATAGGAA	AATCCACCACAACCAAACCT	AGCCCGTATTTCCAACTTCC	Exons 9,10	1267

**7**	CCGAAATGAGGTAGGCAAAC	GCCCTGTCACTCACCCTTAT	-	-	Exons 11	483

**8**	TGGAAACTCACCTCTTTTGTGA	TTCTTTGAGAAGGAAAAGGAAGAA	CAGAGTGGGAGACAAAAGCA	CCAGCTCCTTGGTGTCTCTC	Exons 12,13	1296

**9**	GAAGCAGCAGAAAACCTCCTA	ACCCCACATCCTCTAGCACA	GATCCCCATTTGCATTGTTC	TGGCCCAATTCTTAGGAGTGT	Exons 14,15	889

**10**	GGGTAAAGGATGCGTTTTCA	TGGGAGCTATTATGGGAACC	-	-	Exons 16	332

**11**	TGAGGCTCCAGTTATGGTTCA	CAGTCTTGTTCTCAATCTGCTTC	AAGTGGGACCTAAATGGAAAAG	CCTTTTCCATTTAGGTCCCACT	Exons 17,18	747

**12**	GGATGGGAAAAAGTAGCAAGG	TTCTGATACAAAGAGCCATAGCA	-	-	Exon 19	410

**13**	TTCCCTTTAGTGTTGGGCTTT	TTGGCTTTGGCTAGTCACATT	-	-	Exon 20	92

**14**	GGCTGAATTACCAAAGGTTGT	TTCACAAATCCATCGTCAGG	TCGCAAAATCTGACTTGTGG	GGGAGATGTGCAAGGAATTT	Exon 21, 3'UTR	550

Sequence data were analyzed with ChromasPro v1.5 (Technylisium, Tewantin, Qld, Australia) and compared with the 1.5× poodle (version 1) and the boxer 7.6× whole-genome sequences (CanFam2.0). Among 18 variants identified [14], five variants showed a medium to high disease-associated risk as predicted by FASTSNP [15] and Mutation Taster [16]; three single-nucleotide polymorphisms (SNPs) in a predicted regulatory region of the gene, one synonymous SNP, and a variable sequence repeat in a predicted splice site (Table 2). These variants formed five different haplotypes (Table 3). There were no recombinant events within this 12.6 Kb interval.

**Table 2:** *PTPN22 *sequence variants identified by sequencing genomic DNA from three atopic and three non-atopic WHWTs.

Sequence variant identity	Position on CFA17 (bp)^a,b^	Predicted location in gene	Nucleotide in reference database^b^	Sequence of variant	Reference SNP identity	Predicted functional effect^c^	Variant risk score ^c^	Atopic dogs			Non-atopic dogs			Cross-species conservation of variant nucleotide sequence^b,d^
								**Dog 1**	**Dog 2**	**Dog 3**	**Dog 4**	**Dog 5**	**Dog 6**	

1	54759173	UTR	C	T	rs22597162	Transcription regulatory (score 86.5)	1-3	C/C	C/T	C/T	C/C	C/C	C/C	Conserved in 10/10

2	54759006	UTR	A	del	New variant (dbSNP ss 315790492)	Transcription regulatory (score 87.7)	1-3	del/del	del/A	del/A	T/A	del/A	A/A	Conserved in 9/10

3	54742593	Intronic	A	G	rs22597162	NA	0-2	G/G	G/A	G/A	G/G	G/G	G/G	Not conserved

4	54742027	Intronic	A	T	rs22559551	NA	0-2	T/T	T/A	T/A	A/A	T/A	A/A	Conserved in 6/10

5	54739568	Intronic	T	C	rs22559538	NA	No risk	T/T	C/T	C/T	C/C	C/T	C/C	Not conserved

6	54739315	Intronic	A	G	New variant (dbSNP ss 315790493)	NA	0-2	G/G	G/G	G/G	A/A	A/G	A/A	Not conserved

7	54738923	Intronic	G	del	New variant (dbSNP ss 15790494)	NA	No risk	del/del	del/del	del/del	del/del	del/del	del/del	NA

8	54738927	Intronic	-	A	New variant (dbSNP ss 315790495)	NA	No risk	A/A	A/A	A/A	A/A	A/A	A/A	NA

9	54734456	Intronic	T	C	rs22559532	NA	0-2	C/C	C/T	C/T	C/C	C/C	C/C	Not conserved

10	54734415	Intronic	A	G	rs22559522	NA	No risk	A/A	A/G	A/G	G/G	A/G	G/G	Conserved in 10/10

11	54717953	Exonic	G	A	New variant (dbSNP ss 315790496)	Synonymous Splicing regulatory (score 85.4)	1-4	G/G	G/G	G/G	A/A	A/A	A/A	Conserved in 7/10

12	54715779	Intronic	T	C	rs22578128	NA	0-2	C/C	C/T	C/T	T/T	C/T	T/T	Conserved in 2/10

13	54709793	Intronic (spice site)	17-T repeat (wild)	22-T repeat (variant)	New variant (dbSNP ss 315790497)	Alternative splicing regulatory (score 3.39)	3-4	variant/variant	variant/wild	variant/wild	wild/wild	variant/wild	wild/wild	Conserved in 10/10

14	54699432	UTR	C	T	New variant (dbSNP ss 315790498)	NA	0-2	C/C	C/C	C/C	T/T	T/T	T/T	Not conserved

15	54698793	UTR	G	T	New variant (dbSNP ss 315790499)	NA	1-3	T/T	T/T	T/T	T/T	T/T	T/T	NA

16	54698788	UTR	C	T	New variant (dbSNP ss 315790500)	Transcription regulatory (score 85.4)	1-3	T/T	T/T	T/T	C/C	C/C	C/C	Conserved in 7/10

17	54698729	UTR	T	C	New variant (dbSNP ss 315790501)	NA	1-3	C/C	C/C	C/C	C/C	C/C	C/C	NA

18	54698473	UTR	G	T	New variant (dbSNP ss 315790502)	NA	0	T/T	T/T	T/T	G/G	G/T	G/G	Conserved in 9/10

Sequence variants with a predicted medium to high disease-associated functional effect, with strongly conserved sequence across 10 mammals (dog, human, pig, horse, mouse, rat, cattle, chimpanzee, gorilla and orangutan) and differential distribution between atopic and non-atopic dogs are underlined (Sequence variant identities 1, 2, 11, 13 and 16)

^a^reverse strand; ^b^based on the 1.5× poodle genome (version 1) and the boxer 7.6× whole-genome sequences (CanFam2.0), accessed in March 2010 from http://www.ncbi.nlm.nih.gov and http://genome.ucsc.edu; ^c^as predicted by FASTSNP [5]; disease-risk possibilities are 0 (no potential functional risk), 1 (very low risk), 2 (low risk), 3 (medium), 4 (high risk) and 5 (very high risk); FASTSNP provides a "risk score" for each SNP based on its putative biological function; ^d^analyzed following genomic alignment of flanking regions containing the genetic variants in 10 possible species (dog, human, pig, horse, mouse, rat, cattle, chimpanzee, gorilla and orangutan); UTR: untranslated region (DNA); NA: not accessed; del: nucleotide deletion

**Table 3:** Haplotypes constructed using 18 genetic variants of *PTPN22*

Haplotype^a^		Number of chromosomes	
		
		Atopic dogs	Non-atopic dogs
A	C-del-G-T-T-T-del-A-C-A-C-C-variant^b^-C-T-T-C-T	4/6	0/6

B	T-A-A-C-C-G-del-A-T-G-C-T-wild^c^-C-T-T-C-T	2/6	0/6

C	C-A-G-C-C-A-del-A-C-G-T-T-wild^c^-T-T-C-C-T	0/6	4/6

D	C-A-G-C-C-A-del-A-C-G-T-C-wild^c^-T-T-C-C-G	0/6	1/6

E	C-del-G-T-T-G-del-A-C-A-C-T-variant^c^-T-T-C-C-T	0/6	1/6

^a^maximum-likelihood (Log likelihood = - 108.87) haplotype assignment for the dogs as predicted by Superlink [7]; ^b^22-T repeat allele; ^c^17-T repeat allele; del: nucleotide deletion

**Figure 1:**
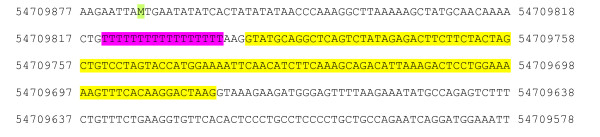
**Relative location of the variant sequence repeat c.2137-20 T(17_22) in canine *PTPN22***. Exons in the gene are marked in yellow, variants annotated in web-based databases are in green and the new intronic variant identified by sequencing in three atopic and three non-atopic WHWTs is highlighted in pink. Line numbering is relative to coordinate system.

Variant sequence repeat c.2137-20 T(17_22) (Figure 1) has not been previously reported in dogs or other species and was bioinformatically predicted to have indirect structural effects on PTPN22. Comparable intronic repeat variations might interfere with normal gene expression [17-19] and have been associated with alternative splicing and disease in humans [20-23]. Thus, fluorescently labelled, amplified-fragment length genotyping of this variant was performed in 72 WHWTs, including 54 dogs from the GWAS. Primers and PCR conditions for amplification of PCR product 11 were used. Genotyping was performed on a 3130xl Genetic Analyzer (Applied Biosystems, Foster City, CA, USA) and analyzed using Genemapper (Applied Biosystems, Foster City, CA, USA). SIB-PAIR [24] showed no significant evidence for allelic association between this variant and the trait (gene-dropping simulation method, *P *= 0.01). Large scale genotyping and complementary genomic and proteomic assays would be required to assess any potential effect of the remaining genetic variants in *PTPN22*.

### Availability of supporting data

The data set supporting the results of this article is available in the National Center for Biotechnology Information Reference Assembly dbSNP repository, http://www.ncbi.nlm.nih.gov/SNP/snp_viewTable.cgi?handle=O_LEARY_ATOPY.

## Competing interests

The authors declare that they have no competing interests.

## Authors' contributions

JBR was responsible for all experimental procedures, analysis and interpretation of data, manuscript writing and editing; CAO conceived and coordinated the study, contributed to the experimental design and to manuscript drafting and editing; MKT contributed to manuscript editing; DLD contributed to the experimental design, statistical analyses and manuscript editing; PG contributed to experimental procedures and analysis of data; LV, KM and MS were responsible for the diagnosis and recruitment of dogs. All authors contributed to the critical revision and approved the final manuscript.
